# Variability of the fimA gene in Porphyromonas gingivalis isolated from periodontitis and non-periodontitis patients

**DOI:** 10.4317/medoral.18042

**Published:** 2012-12-10

**Authors:** Simone Fabrizi, Rubén León, Vanessa Blanc, David Herrera, Mariano Sanz

**Affiliations:** 1 ETEP Research Group, Complutense University, Madrid, Spain; 2 DENTAID S.L, Barcelona, Spain

## Abstract

Objective: The goal of this study was to determine the genetic variability of the fimA gene in Porphyromonas gingivalis isolates from Spanish patients. Study Design: Pooled subgingival samples were taken, processed and cultured in non-selective blood agar medium. Pure cultures of one to six isolates per patient were obtained and PCR and PCR-RFLP were used for fimbrillin gene (fimA) type determination of the extracted genomic (DNA). Results: Two hundred and twenty four Porphyromonas gingivalis isolates from 65 patients were analyzed consisting of 15 non-periodontitis patients (66 isolates) and 50 with periodontitis (158 isolates). Genotype II was the most prevalent (50.9%), while the other types of fimbriae did not exceed fifteen percent of prevalence. Isolates with types II and IV of fimbriae were significantly more prevalent in periodontitis patients than isolates with genotype I. Co-infection was observed in 17.65% of the patients analyzed. Conclusion: The results suggest that in this population Porphyromonas gingivalis with type II of fimbriae are significantly more predominant in periodontitis patients than genotype I.

** Key words:**Fimbriae, genotype, porphyromonas gingivalis, periodontitis.

## Introduction

Porphyromonas gingivalis (*P. gingivalis*) is one of the major pathogens associated with chronic periodontitis. This bacterial species has been frequently isolated in severe forms of chronic and aggressive periodontitis, in sites with severe loss of periodontal attachment, in deep periodontal pockets and in sites demonstrating periodontal disease progression (1). This bacterium has also been associated with other oral infections, such as endodontic infections and periodontal abscesses (2). Moreover, its presence in high numbers in the subgingival biofilm has been associated with different systemic diseases such as cardiovascular disease, pregnancy complications and glycemic disturbances in diabetic patients.

The pathogenicity of these gram-negative anaerobic bacteria has been studied in different experimental animal models. When different strains were inoculated in rats, the effect was highly variable ranging from localized reactions such as the formation of localized abscesses or the exacerbation of periodontal tissue destruction or extreme horizontal and vertical alveolar bone loss, to spreading abscesses leading to the death of the animals (3). These authors reported that a specific group of strains represented by strain type American Type Culture Collection (ATCC) 33277 led to the localized-type abscesses, while another group represented by the W50 and W83 strains, led to the spreading abscess often causing the death of the animals. The high pathogenicity of specific *P. gingivalis* strains has been attributed to the expression of a wide range of virulence factors, such as the external membrane proteins (lipoproteins, carrier proteins), lipopolysaccharide (LPS), fimbriae, proteases (trypsin-protease, collagenase, aminopeptidase, gingipains), lipolytic enzymes and capsular polysaccharides (K-Antigen) (4).

Different *P. gingivalis* strains may also cause indirect tissue damage by stimulating the innate immune response through different pathways. It has been recently demonstrated that different capsular K serotypes of *P. gingivalis* may induce a differential response on human dendritic cells (5) and the antigenic diversity among the different *P. gingivalis* strains (ATCC 33277, W50 and W83) was associated with specific antibody responses (6). This phenotypic and immunological diversity has prompted further investigations aiming to determine *P. gingivalis* genetic variability and its impact on the pathogenicity of this bacterium.

The major fimbriae of *P. gingivalis* have been considered one of the most important virulence factors (7). Fimbriae are filamentous components on the cell surface composed of a subunit protein called fimbrillin. These structures play a major role in the mechanisms of adhesion and invasion of epithelial cells, (8) induce cellular activation and cytokines release (9). For the fimbrillin gene (*fimA*), six different genotypes (*fimA* I, II, III, IV, V and Ib) have been described; and those strains containing genotypes Ib, II, IV and V have shown more virulence, by provoking strong inflammatory reactions in mice, in contrast to types I and III that induced only mild inflammation (10).

Several authors have studied whether the presence of these type-specific *fimA* genotypes of *P. gingivalis* correlate with the prevalence and severity of periodontitis or whether these genotypes are related to different geographical areas or ethnic patient groups (11-16). From these studies, it has been shown that fimA type II is the most prevalent genotype in periodontitis patients followed by types IV, Ib, and V, while *fimA* types I and III are more prevalent in healthy subjects. Most of these studies have been carried out in Japan and few on Caucasian populations (17) what highlights our lack of knowledge on the prevalence of the different genotypes of *P. gingivalis fimA* genes in European populations. Studies published on the prevalence of *P. gingivalis* in Spanish patients reported that a considerable proportion of subjects, irrespective to the periodontal status, harboured this bacterium (18-20). It is therefore the aim of this investigation to study the prevalence of *fimA* genotypes in a sample of patients in this population.

## Material and Methods

-Subjects

The sample population consisted on consecutive patients attending the postgraduate clinic for periodontics at the complutense university of Madrid, Spain.

Subjects had a conventional whole-mouth periodontal examination using a North Carolina periodontal probe (Hu-Friedy Mfg. B.V., Rotterdam, The Netherlands) and a radiographic exam with periapical and/or panoramic X-rays. This clinical and radiological data, combined with the patient’s medical history allowed the categorization of these subjects using internationally accepted diagnostic criteria (21) as chronic and aggressive periodontitis patients, or non-periodontitis.

Subjects were selected for this microbiological investigation if they were systemically healthy, had a minimum of 16 teeth (at least 3 per quadrant), have not undertaken any periodontal therapy and were not on any relevant medication (no previous antibiotic medication at least one month prior to entering the study).

-Microbiological sampling 

In these subjects, pooled samples were taken from four periodontal sites, one per quadrant corresponding with the deepest probing depths that bleed on probing during the periodontal examination. At these sites, two consecutive sterile paper points (Fine, West Palm Beach, CA, USA) were inserted into the depth of the pocket and left in place for 10 seconds (s). All the paper points were pooled in a vial containing 1.5 ml of reduced transport fluid (RTF) and immediately transported to the laboratory, where samples were processed within 1 hour.

-Microbiological procedures

At the laboratory, samples were vortexed and diluted in buffer phosphate saline (10, 100, and 1000 times). An aliquot of 100 µL was placed on culture plates containing non-selective blood agar medium (Oxoid Nº 2; Oxoid, Basingstoke, UK), supplemented with 5% horse blood, haemin (5 mg/L) and menadione (1 mg/L). They were then incubated at 37ºC for 7 days in anaerobic conditions. Colonies of *P. gingivalis* were identified by its characteristic morphology and was further confirmed by gram-staining and with the API 32A system (Biomerieux, Le Balme Les Grottes, France).

Only the subjects that were *P. gingivalis*-positive after anaerobic culturing were included in this investigation. One to five colonies from each patient were selected to obtain pure cultures that were stored at -80 °C for further analysis.

-Genomic deoxyribonucleic acid (DNA) extraction and purification 

Total genomic DNA was extracted from 224 isolates, following the method described by Sambrook and Russel in 2001. Briefly, the cells growing in brain heart infusion (BHI) medium, supplemented with haemin and menadione were centrifuged (Hettich, Universal 320R centrifuge) and washed with Solution I. They were then re-suspended in 1 ml of Solution I with lysozime (10 mg/ml) and incubated during 10 min at 37°C. Proteinase K (1.0 mg/ml), ribonuclease (RNase) (20mg/ml) and sodium dodecyl sulphate (SDS) were then added, and incubated at 37°C until total cell lysis occurred. After several steps of extraction with phenol: chloroform: isoamyl alcohol (25:24:1) and chloroform: isoamyl alcohol (24:1), nucleic acids were precipitated with absolute ethanol and re-suspended in buffer Tris-HCl (TE) 10 mM, ethylenediaminetetraacetic acid (EDTA) 1,0 mM pH 8,0. Genomic DNA and polymerase chain reaction (PCR) products were analyzed using agarose gel electrophoresis in 1X Tris-acetate-EDTA (TAE) buffer and visualized by staining with ethidium bromide.

-fimA typing in P. gingivalis isolates

fimA type determination was done by PCR using pri-mers previously reported (12,22,23). PCR assays were performed in a thermal cycler (TECHNE TC-412). After denaturation at 96 ºC for 3 min, a total of 35 PCR cycles were performed; each cycle consisting of 30 s of denaturation at 95 ºC, 30 s of annealing at 55 ºC and 180 s of extension at 72 ºC.

**Figure 1:**
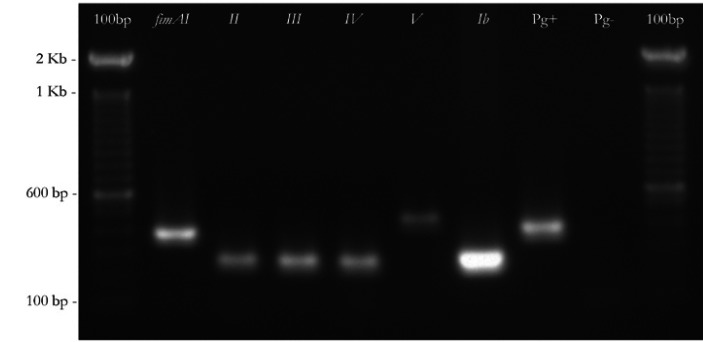
Detection of the six different types of *fimA* genes of *P. gingivalis*, by Polymerase chain reaction (PCR), isolated from the patients analyzed in this study. From the left to the right: 100 base pair (bp). Molecular weight size marker; *fimA* type (bp). I (392), II (257), III (247), IV (462) and Ib (271); *P. gingivalis* (Pg). Ribosomal ribonucleic acid rRNA fraction; Pg-. Negative control.

**Figure 2:**
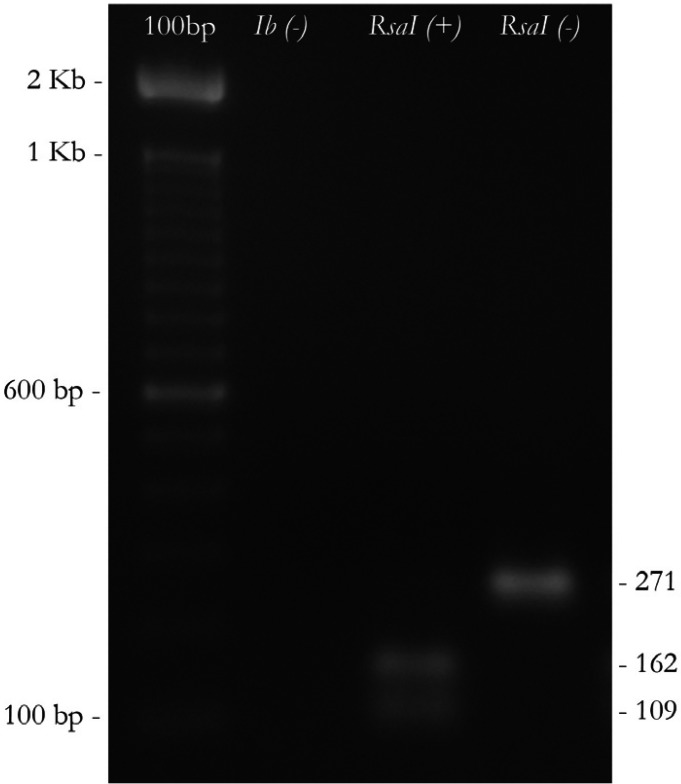
Differentiation of the Ib genotype using PCR-Restriction fragment length polymorphism (PCR-RFLP). Digestion was obtained by a restriction enzyme from the bacterium Rhodopseudomonas sphaeroides (RsaI). From the left to the right:100bp. Molecular weight size marker; (Ib-). *fimA* type II; (RsaI+). *fimA* type Ib. (RsaI-). *fimA* type I.

Following the method described by Nakagawa et al. (12), in the isolates that simultaneously reacted with primers of type I and type II fimA, another PCR amplification was carried out with type Ib-F and Ib-R primer pairs, after which the amplified fragments were digested by RsaI enzyme to distinguish *fimA* type Ib from *fimA* type I. In figure 1 and figure 2 the amplification pattern is shown for all *fimA* genotypes, using our clinical isolates. *P. gingivalis* (ATCC 33277 (*fimA* I), Hg184 (*fimA* II), W50 (*fimA* IV), 49417 (*fimA* III), HG1690 and HG1691 (*fimA* Ib)) were used as controls.

-Data analysis

The association between each *fimA* genotype and the patient’s periodontal status (non-periodontitis versus periodontitis) was studied at both patient and isolate levels.

At a patient level (65 patients), the association between the clinical status and the type of fimbriae was depicted by contingency tables and evaluated using Chi-square (χ2) tests. In addition, differences between the distribution of clones with type I fimbriae versus other types of fimbriae in both groups were tested by Fisher’s Exact Test, based on the previously reported associations of type I with health and types II, IV and Ib with periodontitis (24). Finally, the odds ratio (OR) for a patient harbouring one of the six types of fimbriae, belonging to the periodontitis group was calculated.

At isolate level (224 isolates), the association between the clinical status and the type of fimbriae was assessed by means of a χ2 tests, in contingency tables. In addition, as explained before, differences between the distribution of clones with type I fimbriae versus the distribution of clones with other types of fimbriae in both groups were tested by Fisher’s Exact Test. Finally, we calculated the OR for a clone with one of the six types of fimbriae, of being isolated in the group of periodontitis patients.

## Results

 Table 1 depicts the frequency of detection of the different *P. gingivalis fimA* genotypes, among all the evaluated isolates (n=224). Overall, the most frequent genotype was type II (50.9%), while none of the other *fimA* genotypes represented more than 15% of the isolates.

**Table 1:**
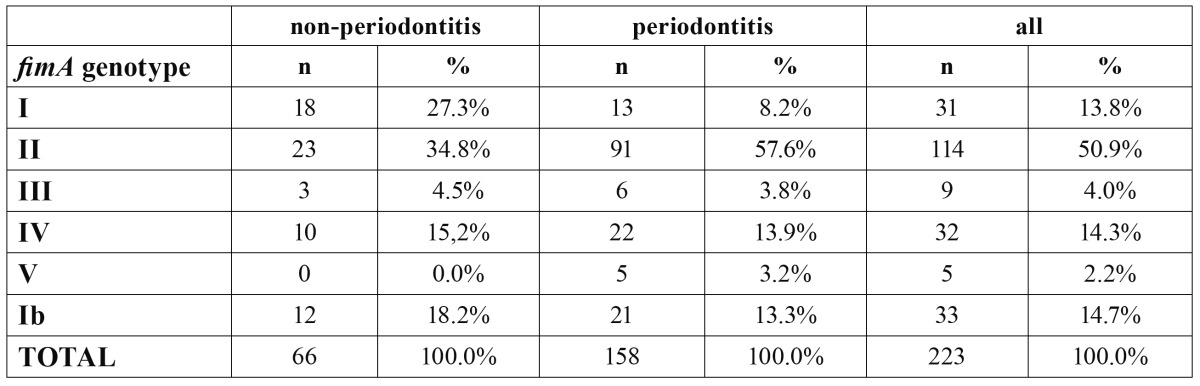
Frequency, as absolute number (n) and proportion per group (%), of different *fimA* genotypes in isolates from non-periodontitis or periodontitis patients.

When comparing the isolates from periodontitis versus non-periodontitis subjects, statistically significant differences were detected (p=0.001). Specifically, *fimA* genotype II was detected more frequently in isolates from periodontitis subjects (57.6% versus 34.8%); whereas, *fimA* genotype I was detected more frequently in isolates from non-periodontitis subjects (27.3% versus 8.2%). The rest of the fimbriae genotypes were detected in low numbers.

The distribution of clones with type I fimbriae was compared with the distribution of clones with other types of fimbriae, in both groups. Both types II (p<0.001, OR=2.878) and IV (p=0.044, OR=1.858) fimbriae were significantly more prevalent in patients with periodontitis, when compared to genotype I.

 Table 2 depicts the prevalence of the different fimbriae type, or combinations of types, among the 65 subjects studied. Overall, *fimA* genotype II alone was the most frequent category (46.2%) while *fimA* genotype V was the least prevalent (1.5%). No other category represented a proportion higher than 13%. Genotype II was significantly more prevalent than genotype I in the total sample (p=0.024).

**Table 2:**
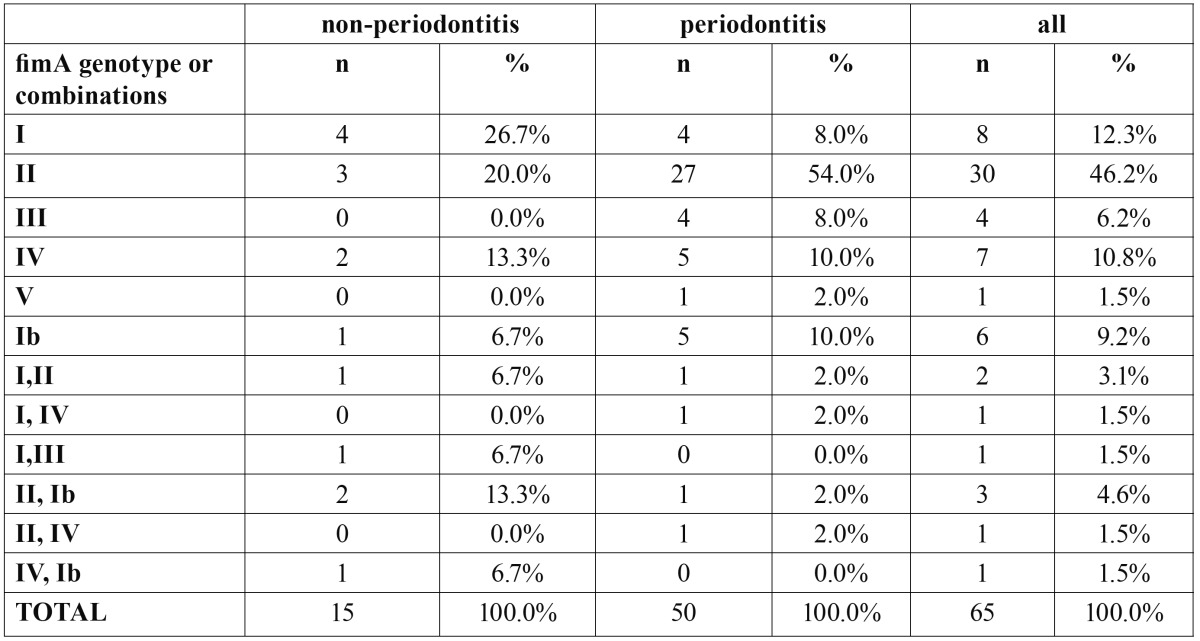
Frequency, as absolute number (n) and proportion per group (%), of patients with different *fimA* genotypes, or combinations, in both non-periodontitis or periodontitis groups.

In periodontitis subjects, the most prevalent fimbriae was genotype II (54%), while in non-periodontitis subjects were genotypes I (26.7%) and II (20%). Differences among groups showed a tendency towards statistical significance (p=0.005). Patients with type II fimbriae had a significantly higher risk (p=0.025, OR=5.0) of suffering periodontitis, than those harbouring genotype I.

Since 51 out of 65 patients provided more than one isolate, an evaluation of “co-infection” was possible. More than one *fimA* genotype was found in 9 subjects (5 non-periodontitis subjects and 4 periodontitis patients). The occurrence of monoclonal infection (only one genotype per patient) was significantly more likely in periodontitis versus non-periodontitis subjects (p=0.025). Periodontitis patients had less risk of co-infection (OR=0.321).

## Discussion

The results from the present investigation in *P. gingivalis* isolates from Spanish subjects with a predominant Caucasian population showed that the most frequent fimbriae genotype was *fimA* II (46.2%). This fimbriae type was the most prevalent among all the isolates studied (50.9%) and also the most frequent type among periodontitis patients (54.0%), being this difference statistically significant when compared to non-periodontitis subjects.

These results are in agreement with previous studies (17,22,25,26) reporting that *fimA* genotype II was associated with periodontitis and thus confirming that the distribution of the different *P. gingivalis* fimbriae may not vary between different ethnic groups or geographical areas. In the referred studies, it was reported that genotypes IV and Ib were the second most prevalent in patients with periodontitis. Our study could not confirm these finding, since genotypes IV and Ib in our sample were almost equally distributed among non-periodontitis and periodontitis patients. Conversely, genotypes I and III have been described as the most prevalent in healthy patients (11,26,27). Our results found that *fimA* I was the most frequent genotype in non-periodontitis subjects, but genotype III isolates were equally distributed in both groups.

Recent prospective studies have shown that the initiation and progression of periodontitis in young Moroccan subjects was associated with the presence of a highly pathogenic clone (leukotoxic JP2 clone) of Aggregatibacter actinomycetemcomitans (A. actinomycetemcomitans) species (relative risk 18.0; 95% CI 7.8–41.2; p<0.0001) (28). However, for *P. gingivalis*, although in vitro studies have demonstrated a high degree of variability in the genes responsible for the expression of some of the virulence factors, the clonal association with the initiation and progression of periodontitis has not been demonstrated. Among the widely studied virulence factors, the major fimbriae is one of the most important, and several studies have described the variability of the *fimA* gene (12,22,23) and the frequency of the different genotypes in isolates from subjects of different ethnic groups and geographic locations (24). In the present investigation, the frequency distribution of isolates from the different *fimA* genotypes was: type II (50.8%), type I (13.0%), type IV (14.2%) and type Ib (14.7%). When comparing these results with those from previously published investigations, there is a general agreement that genotypes I, II and IV are the most frequently detected, regardless of the subject’s periodontal condition (14,16,17,27).

Our data showed a low frequency of co-infection with more than one clone (4.5%); this distribution disagrees with what was reported in Asian populations (9.5%-23%) and in Caucasian populations (27) where the reported percentages of co-infection were higher than 70%. These differences in the latter studies may be due to the use of techniques with higher sensitivity, thus allowing higher detection (25). In our study, the number of patients enrolled and the number of isolates per patient are insufficient to determine the prevalence of co-infection in our population.

The findings of the present study agree with the results from in vitro studies where the virulence of type I strain (ATCC 33277), a type II strain (OMZ314) and two mutants originating from these strains (for which the *fimA* type I gene was substituted by type II and vice versa) were examined (29). Data from these investigations showed that strains containing fimbriae type II had a greater capacity to adhere to and invade epithelial cells, to inhibit migration and cellular proliferation and to cause abscesses in a mice model. In addition, when the capacity of these strains to form biofilms has been studied in vitro, it was observed that *P. gingivalis* with type II fimbriae tend to form abundant biofilms with clustered colonies, and those containing type I tend to form biofilms with more dispersed microcolonies and reduced volumes.

Studies using multilocus sequence typing method (MLST) of *P. gingivalis fimA* gene recommend re-evaluating the genotyping methods used for this gene. They observed that by MLST analysis it is possible to observe a wider genetic diversity and also to prevent false positive results, especially when PCR is performed directly from clinical material (13,27). In our work we obtained pure cultures of each isolate and they were tested with all primer pairs achieving very clear results.

In addition, it has been suggested that studying the variability of other virulence factors, such as gingipains, capsule or minor fimbriae and their joint action will allow for more accurate assessment of the virulence levels for each strain (26,30). Nevertheless, the different epidemiological studies carried out in different types of patients in Japan, China, Norway, Germany, Brazil, among others; consider *fimA* type II gene as one of the most important virulence factors of *P. gingivalis* which is likely related to the pathogenesis of periodontitis.

Within the limitations of the present study, the reported data demonstrated a significant association between the presence of the *fimA* genotype II and periodontitis, and underline the need of further studies with larger sample populations, more sensitive genotyping methods and genotyping methods able to study several virulence factors at the same time.
